# Neuromyotonia with polyneuropathy, prominent psychoorganic syndrome, insomnia, and suicidal behavior without antibodies: a case report

**DOI:** 10.1186/s13256-015-0581-0

**Published:** 2015-05-06

**Authors:** Edvard Ehler, Alena Meleková

**Affiliations:** Neurology Clinic, Pardubice Regional Hospital and Faculty of Health Studies, University of Pardubice, Kyjevská 44, 532 03 Pardubice, Czech Republic

**Keywords:** Insomnia, Myokymia, Neuromyotonia, Polyneuropathy, Suicidal behavior

## Abstract

**Introduction:**

Peripheral nerve hyperexcitability disorders are characterized by constant muscle fiber activity. Acquired neuromyotonia manifests clinically in cramps, fasciculations, and stiffness. In Morvan’s syndrome the signs of peripheral nerve hyperexcitability are accompanied by autonomic symptoms, sensory abnormalities, and brain disorders.

**Case presentation:**

A 70-year-old Caucasian man developed, in the course of 3 months, polyneuropathy with unpleasant dysesthesia of lower extremities and gradually increasing fasciculations, muscle stiffness and fatigue. Subsequently, he developed a prominent insomnia with increasing psychological changes and then he attempted a suicide. Electromyography confirmed a sensory-motor polyneuropathy of a demyelinating type. The findings included fasciculations as well as myokymia, doublets and multiplets, high frequency discharges, and afterdischarges, following motor nerve stimulation. No auto-antibodies were found either in his blood or cerebrospinal fluid. Magnetic resonance imaging of his brain showed small, unspecific, probably postischemic changes. A diagnosis of Morvan’s syndrome was confirmed; immunoglobulin (2g/kg body weight) was applied intravenously, and, subsequently, carbamazepine 2×200mg, venlafaxine 150mg, and mirtazapine each night were prescribed. His sleep improved, suicidal tendencies stopped, less fasciculations occurred, and muscle hypertonia also improved. Hyperexcitation also partially remitted including the electromyography finding.

**Conclusions:**

We described here the case of a patient with Morvan’s syndrome; his case is rare because of severe psychical changes with a suicide attempt, short admission to a psychiatric ward, prominent electromyographic changes, and because antibodies were not detected. After therapy with immunoglobulins followed by corticosteroids with sodium channel blocker, his motor, autonomic, psychical signs and symptoms, and electromyography changes substantially improved.

## Introduction

Peripheral nerve hyperexcitability disorders are characterized by constant muscle fiber activity due to hyperexcitability in the distal motor axons [1]. Acquired neuromyotonia is characterized by continual ectopic nerve activity, which manifests clinically in cramps, fasciculations, and stiffness. These symptoms are accompanied by autonomic symptoms, sensory abnormalities, and, in the case of Morvan’s syndrome, by brain disorders. Apart from neuromyotonia, Morvan’s syndrome manifests central symptoms (insomnia, hallucinations, anxiety, agitation, confusion), autonomic symptoms (hyperhidrosis, tachycardia, obstipation) [2]. On neurophysiological examination, neuromyotonia manifests prominent spontaneous activity: fibrillations, positive waves, fasciculations, myokymia, multiple discharges, neuromyotonic discharges, doublets and multiplets. After voluntary contraction, and after electric stimulation of motor fibers, multiple and long-lasting afterdischarges occur [2]. There is no clear consensus as to the part of the peripheral motor neuron in which this ectopic activity arises. Most authors locate the ectopic focus in distal terminal motor fibers. Both antidromic propagation of excitation and axon reflex can participate in triggering this ectopic activity. In some conditions (inflammatory changes of the central nervous system; CNS) ectopic activity sites appear in the area of the anterior horn of the spinal cord as well as in higher levels of the CNS [3].

## Case presentation

This case report describes a 70-year-old Caucasian man with presented, and electrophysiologically confirmed, neuromyotonia with significant autonomic and psychological changes (insomnia, anxiety, suicidal behavior), and subsequent successful treatment.

In April 2014, the 70-year-old man developed crural and leg pain, chills, tingling, hypersensitivity to mechanic stimuli, and slight weakening of lower limbs with mild foot-drop. This disorder developed quickly and no trigger was identified. In June 2014, he developed insomnia, anxiety, confusion, even auditory hallucinations, and he attempted suicide by slashing his left wrist. For a short period of time, he was admitted to a psychiatric ward.

**Figure 1:**
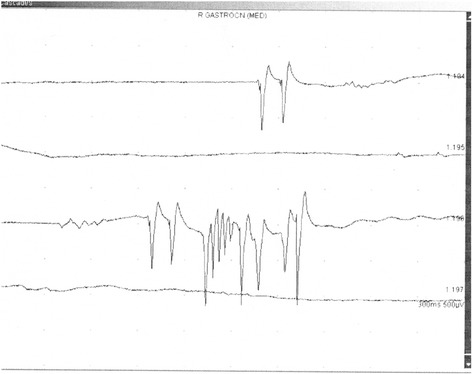
Neuromyotonic discharge in musculus gastrocnemius medialis; right.

**Figure 2:**
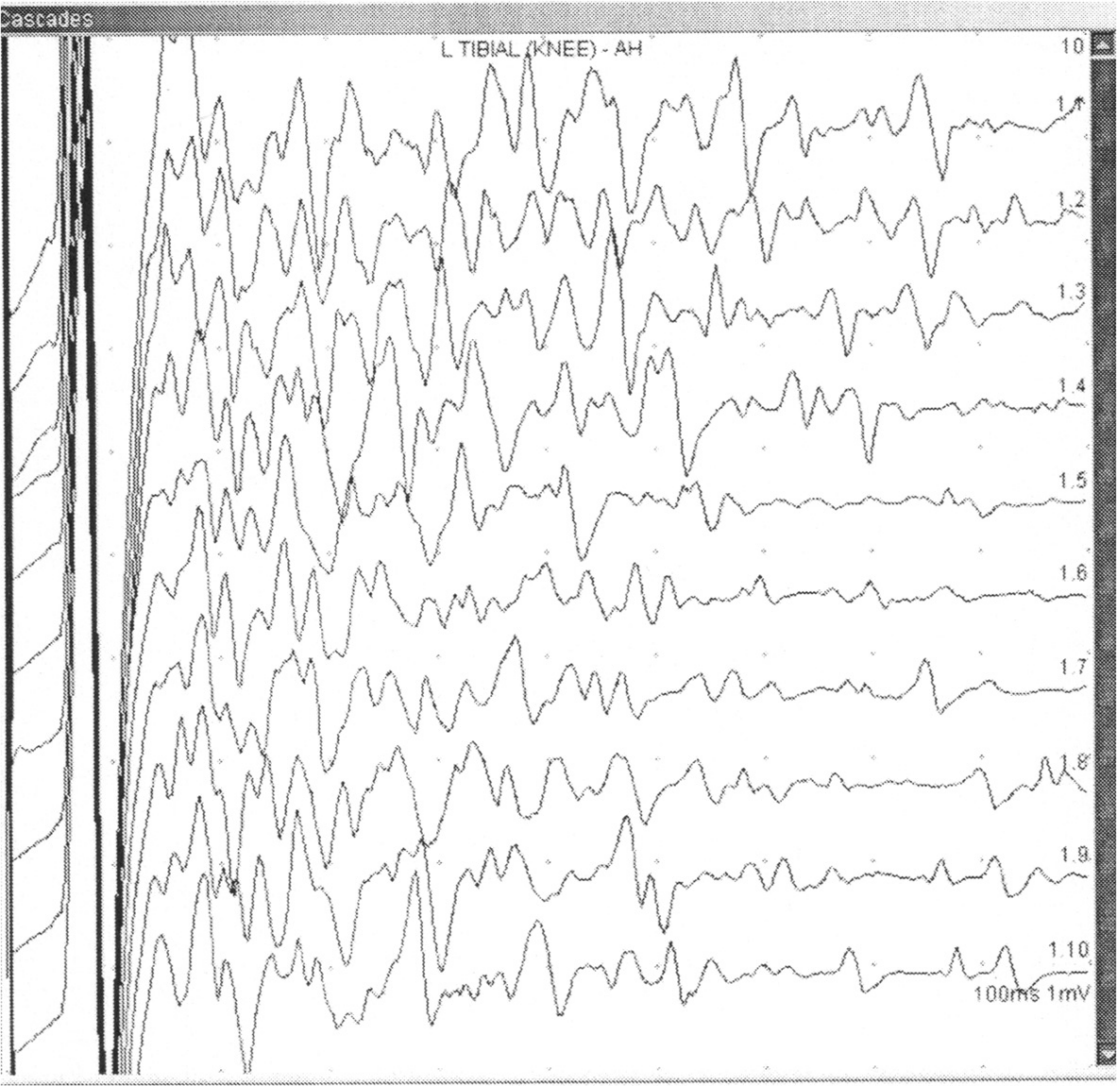
High voltage and long-lasting afterdischarges F-wave study in tibial nerve; left.

He was referred to our neurological clinic electromyography (EMG) laboratory because of muscle weakness with prominent fasciculations, myokymia, and suspected amyotrophic lateral sclerosis. An EMG confirmed fibrillations, positive waves, fasciculations, and multiple myokymic and neuromyotonic discharges, occasional high frequency fasciculations, doublets and multiplets (Figure 1). Contraction curve was reduced with unstable motor unit potentials with neurogenic changes. A motor nerve conduction study showed multiple afterdischarges with long-lasting repetitions, which prevented F-waves assessment (Figure 2). His grip strength was weakened. He could briefly stand on tiptoes but only with difficulties; walking on heels was only barely attempted. He also presented tactile, thermic and vibratory hypesthesia of his lower and upper limbs.

His blood test showed high levels of creatine kinase (CK) 12.26 (nkat/L; normal value 3.60), CK myocardial bound 0.52, but no other abnormalities. No antibodies (contactin associated protein-like 2, CASPR2; leucine-rich glioma inactivated 1 protein; contactin 2; anti-glutamic acid decarboxylase) were found in his blood or in his cerebrospinal fluid (CSF). Oligoclonal protein synthesis was not confirmed. Cancer was not detected (oncological markers were negative; the results of chest X-ray, ultrasonography of abdomen, endoscopic investigation of gastrointestinal tract and urological examination were negative). His CSF showed borderline results: protein 0.40g/L and 1 mononuclear cell/1mm^3^.

The findings were evaluated as neuromyotonia associated with central cerebral symptoms consistent with Morvan’s syndrome. No antibodies (including voltage-gated potassium channel, VGKC, and CASPR2) were found either in his blood or CSF.

For a period of 5 days, immunoglobulin was applied intravenously at a dosage of 0.4g/kg body weight. A rapid improvement in his muscle strength occurred, his fasciculations decreased, and his pain sensation disorders were alleviated, including hyperalgesia. Subsequently, venlafaxine 150mg, carbamazepine 2×200mg, and mirtazapine 30mg each night were prescribed. Both his sleep disorder and daytime fatigue were alleviated. A follow-up EMG showed an increased A-sensory nerve action potential and shortened duration of afterdischarges. His blood CK level decreased to 2.31nkat/L. He was discharged after 14 days. A follow-up examination confirmed stable state; he had no sleep disorder, hallucinations or depression.

The findings were assessed as Morvan’s syndrome of an autoimmune type with undetected specific antibodies.

He has been treated with prednisolone and carbamazepine for more than 8 months and is still without substantial cramps, profuse sweating and psychical problems. We expect from the facts that his outcome will be good [2,4].

## Discussion

We described here the case of a 70-year-old man with Morvan’s syndrome; his case is rare because of his severe psychical changes (a suicide attempt and short admission to a psychiatric ward), prominent electromyographic changes, and because antibodies were not detected. In patients with Morvan’s syndrome without antibodies their therapy should begin with intravenous immunoglobulins (IVIGs) followed by corticosteroids and with sodium channel blocker. After this recommended therapy the motor, autonomic, psychical signs and symptoms, and some of the neurophysiologic changes in our patient substantially improved.

An acquired neuromyotonia is frequently associated with autoimmune disorders, thymus cancer, lymphoma, or lung cancer. Morvan’s syndrome is characterized by neuromyotonia, prominent autonomic symptoms (hyperhidrosis, obstipation, and tachycardia) and cerebral symptoms: insomnia, agitation, anxiety, confusion, and hallucinations. Patients with Morvan’s syndrome display prominent leg pain, which sometimes becomes a burning sensation, and myalgia [4]. In Morvan’s syndrome, antibodies against VGKCs are confirmed. These antibodies cause a reduction in the number of potassium channels, and thus prolong nerve action potential on the axon membrane [3]. Antibodies against VGKC occur in hyperexcitability of the peripheral nerve, as well as in limbic encephalopathy, epileptic seizures, and myoclonus; that is the reason why some authors use the term “VGKC syndromes”. Antibodies are found in 30 to 50% of patients with neuromyotonia and in the majority of patients with Morvan’s syndrome [5]. We failed to detect any antibodies or tumor in our patient. Also other authors describe patients with Morvan’s syndrome of autoimmune origin without detection of VGKC. Our diagnosis was based on clinical findings, medical history, and predominantly on neurophysiological findings.

Therapy of Morvan’s syndrome is focused on suppression of antibody production, influencing ion channels, and, subsequently, neuropathic pain control. In patients with positive findings of VGKC antibodies it is recommended to commence the therapy with plasmapheresis with subsequent oral immunosuppressive therapy applying corticoids or azathioprine [5]. In patients without VGKC antibodies plasmapheresis is not efficient, and it is recommended to start with a full dose of IVIG (2g/kg body weight), and then continue with a maintenance dose of corticoids [3]. Carbamazepine at a dose of 400 to 600mg per day leads to disappearance of fasciculations and neuropathic pain [2].

## Conclusions

Morvan’s syndrome is characterized by motor symptoms (neuromyotonia), neuropathic pain, autonomic dysfunction and cerebral symptoms (including insomnia and confusion). Clinical presentation and neurophysiological findings are essential for diagnosis determination, whereas VGKC antibodies are detected only in 50% of cases. In Morvan’s syndrome without detected antibodies, it is efficient to apply IVIG with subsequent oral therapy by corticosteroids and potassium channel blockers to reduce autonomic symptoms and neuropathic pain. An efficient therapy significantly alleviates a patient’s complaints and clinical findings (fasciculation, pseudomyotonia), as well as improvement of typical changes detected by neurophysiological examination (myokymia, discharges, afterdischarges).

We described here a case of a 70-year-old man with severe Morvan’s syndrome; his case is rare because of prominent psychical changes (short hospitalization in psychiatric ward, suicide attempt), severe neurophysiological findings, and because he had no antibodies. After immunomodulation therapy his clinical and electrophysiological findings partially improved. He was recommended to our EMG lab with suspicion of amyotrophic lateral sclerosis because of the prominent fasciculations in his muscles and weight loss.

## Consent

Written informed consent was obtained from the patient for publication of this case report and accompanying images. A copy of the written consent is available for review by the Editor-in-Chief of this journal.

## Abbreviations

CASPR2: Contactin associated protein-like 2
CK: Creatine kinase
CNS: Central nervous system
CSF: Cerebrospinal fluid
EMG: Electromyography
IVIG: Intravenous immunoglobulin
VGKC: Voltage-gated potassium channel
